# Reflections on the Origin of Coded Protein Biosynthesis

**DOI:** 10.3390/biom14050518

**Published:** 2024-04-25

**Authors:** Juan Carlos Fontecilla-Camps

**Affiliations:** Univ. Grenoble Alpes, CEA, CNRS, IBS Metalloproteins Unit, F-38000 Grenoble, France; juan.fontecilla@ibs.fr

**Keywords:** RNA polymerase, aminoacyl-tRNA synthetase, ribosome, substrate-assisted catalysis, entropic trap

## Abstract

The principle of continuity posits that some central features of primordial biocatalytic mechanisms should still be present in the genetically dependent pathway of protein synthesis, a crucial step in the emergence of life. Key bimolecular reactions of this process are catalyzed by DNA-dependent RNA polymerases, aminoacyl-tRNA synthetases, and ribosomes. Remarkably, none of these biocatalysts contribute chemically active groups to their respective reactions. Instead, structural and functional studies have demonstrated that nucleotidic α-phosphate and β-d-ribosyl 2′ OH and 3′ OH groups can help their own catalysis, a process which, consequently, has been called “substrate-assisted”. Furthermore, upon binding, the substrates significantly lower the entropy of activation, exclude water from these catalysts’ active sites, and are readily positioned for a reaction. This binding mode has been described as an “entropy trap”. The combination of this effect with substrate-assisted catalysis results in reactions that are stereochemically and mechanistically simpler than the ones found in most modern enzymes. This observation is consistent with the way in which primordial catalysts could have operated; it may also explain why, thanks to their complementary reactivities, β-d-ribose and phosphate were naturally selected to be the central components of early coding polymers.

## 1. Introduction

A central issue in the “origin of life” field concerns the mechanisms in action at the origin of biocatalysis and the nature of the first catalyst(s). The “RNA world” hypothesis posits that abiotically formed RNAs, which could replicate, acquired catalytic properties and became ribozymes with multiple activities [1]. Although the “RNA world” concept has had a major impact in the field, recently, several authors have discussed the implausibility of life solely relying on ribozyme-catalyzed reactions [2,3,4]. The reasons are multiple: a limited number of available catalytic groups, the general instability of the RNA polymers, and the likely impossibility of generating their own ancestors reliably.

Conversely, if the first catalysts were metal ions and/or mineral surfaces, a combination of autocatalytic metabolic cycles would have progressively generated a variety of sugars, amino acids, and nucleobases, later co-evolving to form our “nucleic acid-protein” world. Several authors have discussed primordial nucleic acid–peptide interactions (see, for instance, [3,5,6,7]), and the synthesis of a number of different RNAs and polypeptides on mineral surfaces is well documented [8,9,10,11].

Here, we address three bimolecular reactions generally considered to be very ancient, essential for protein synthesis: ribosomal peptide formation, tRNA aminoacylation, and RNA polymerization. It is concluded that primordial biocatalysis was most probably assisted by substrate groups, such as ribose and phosphate, and by entropically favored binding sites, without the direct involvement of the biocatalyst’s reactive functions. Interestingly, phosphoribosyl pyrophosphate (PRPP), a plausible precursor of early coding nucleotidic polymers, can be readily synthesized from ribose and phosphate on a fumed silica surface [12].

## 2. Background

### The First Functional Coded Polypeptides

Although the first functional polypeptides are often considered to have been uncoded [13,14,15], this is rather problematic. Besides the fact that uncoded peptides can constitute a very vast array of combinations (a ten-residue-long peptide based on four dissimilar aminos acids can potentially have about one million different sequences), they are not subjected to natural selection in the Darwinian sense. This is because there would be no way to reliably reproduce a functionally advantageous peptide. In addition, to propose that functional random peptides could have been involved in the triplet genetic code evolution [13] may be complicated because it does not agree with the principle of continuity. In 1968, Francis Crick discussed two possibilities for the origin of the genetic code: either it resulted from a series of “frozen accidents” or it had an stereochemical basis, meaning that some amino acids could have specifically interacted with nucleotide triplets [16]. Although he seemed to favor the first option, he also found it “too accommodating”. F. Crick concluded that the stereochemical hypothesis made more sense and that further experiments should be run to test it [16].

In fact, there are now experimental data that support the notion that, at the origin of life, abiotically generated random RNA sequences could have *selectively* bound a series of amino acids that might have conferred them increased stability. Indeed, Yarus et al. [17] and Johnson & Wang [18] have reported specific interactions between anticodon-containing RNAs and their cognate amino acids. In the case of His, Ile, Phe, Trp, and Tyr, in vitro selection (SELEX) experiments indicated that the joint probability for their binding to a cognate aptamer’s anticodon to be a random process is vanishingly small, with a calculated value of 2.1 × 10^−46^ [17]. Johnson & Wang [18] carried out a distance-based structural analysis on the ribosome and found that the side chains of Asp, Leu, Ile, Arg, Gln, His, Lys, Phe, Tyr, Met, and Trp from riboproteins are frequently located near their cognate anticodons in ribosomal RNAs (rRNAs). Furthermore, in an in silico study, Krüger et al. found that Asn, Leu, Arg, Gln, His, Lys, Phe, and Tyr interacted with several selected RNAs (complete ribosomes were not included in their analysis) [19].

Thus, for many amino acids, the preferred RNA interactions appear to be with their corresponding anticodon-containing sequences. However, even an initially less-specific binding could have been evolutionary advantageous [20]. If several amino acids interacting with a singled-stranded RNA (ssRNA) molecule were at the right distance to form peptide bonds with their neighbors, they could have condensed on a mineral surface, generating a “coded” polypeptide still bound to the oligonucleotide [17]. Even if that ssRNA was minimally stabilized by the resulting polypeptide, which also could have marginally promoted its replication and subsequent melting, it would have replicated more reliably and more often than other, less-stable ssRNAs. Furthermore, if its complementary strand also generated an equivalent functional polypeptide [21], these RNA–polypeptide complexes would have been poised, maybe for the first time, to evolve in a Darwinian sense. Mutual stabilizing interactions have been reported for duplex RNAs and depsipeptides containing positively charged Arg, Lys, and His residues [22].

If this reasoning is correct, the smaller Ala, Ser, Pro, Thr, Gly, and Val would have been excluded from the first functional coded polypeptides because they did not interact strongly enough with RNA [20]. Ala, Ser, Pro, and Thr have NGN anticodons, and Gly and Val have NCC and NAC anticodons, respectively, whereas the RNA-interacting Asn, Leu, Lys, Phe, and Tyr have one anticodon consisting of A/U nucleotides, and Ile has two. Based on these observations, we have suggested that the initial genetic code had only eight (anti)codons composed of A and/or U [20]. Consequently, the first RNAs would have been poly(A/U) oligonucleotides, and C and G would have been added later [23], as the genetic code evolved. An initial code based on triplets combining two different nucleobases was already considered by F. Crick [16]. In any case, a polypeptide-coding system must have appeared very early in the evolution of life, so that natural selection could begin to operate.

## 3. The First Biocatalysts

Extant enzymes come in several distinct groups, but the major factors that contribute to catalysis are the following: (i) entropy effects and orbital steering (optimal orientation so that the transition state is readily formed); (ii) some enzymes may form unstable covalent intermediates that more readily react to form products; (iii) the enzyme may provide proton acceptor and donor groups for general acid-base catalysis; (iv) substrate binding may result in the strain or distortion of the bond targeted to be broken; and (v) some active site groups are positioned to stabilize a reactive intermediate (“propinquity” effect, in [24]). In addition, by not stabilizing or even destabilizing the transition state of an undesired product, the active site can direct the reaction towards a less-preferred option in solution (“negative catalysis”) [25]. Below we will discuss which of these factors may apply to early biocatalysis.

As suggested above, besides stabilizing the oligonucleotide [22], some of the first coded polypeptides could have had proto-RNA polymerase and proto-helicase catalytic activities. If the notion that proto-cells first evolved on a mineral surface [26] is correct, when they eventually detached from that surface, the reactions responsible for amino acids and nucleotides’ polymerization had to rely on primordial soluble biocatalysts. Although it is clear that peptide synthesis must have significantly evolved since then, contemporary ribosomes, aminoacyl-transfer(t)RNAs synthetases, and RNA polymerases should give us some useful indications about the reactions catalyzed by their very early counterparts.

### 3.1. The Ribosome

Protein synthesis is a complex process involving three major stages: initiation, elongation, and termination [27]. Here, we will discuss the mechanism of peptide bond synthesis that takes place in the ribosomal peptidyl transfer center (PTC). Several X-ray structures of the 50*S* and 30*S* prokaryotic ribosome subunits [14,28], as well as that of the 70*S* whole organelle [29], have provided many important insights regarding the catalyzed reaction and its evolutionary origin. Although the ribosome contains several proteins, the PTC is buried in the large 50*S* RNA subunit, almost 18 Å away from any protein chain [14]. The corresponding small 30*S* subunit binds the messenger RNA and places two contiguous codons in a position suitable to interact with their two cognate anticodon loops from respective tRNAs bound to the 50*S* subunit [29].

In 2003, Moore, Steitz, et al. proposed that the observed catalytic effect was partly enhanced by the correct alignment of the bound tRNAs—often called “substrate juxtaposition” or “propinquity” [24]—and, partly, by a basic group that should increase the nucleophilicity of the attacking α-amino group [28]. In 2004, Sievers et al. compared the rate of uncatalyzed peptide formation—in the reaction of the ethylene glycol ester of *N*-formylglycine with Tris(hydroxylmethyl)aminometane—with the rate of peptidyl transfer by the ribosome using NMR [30]. These authors also determined the respective activation parameters and concluded that the observed 2 × 10^7^ rate enhancement in the ribosomal reaction was entirely due to the lowering of the entropy of activation (in fact, the enthalpy of activation was slightly less favorable for the ribosome). Indeed, the entropy of activation of peptidyl transfer within a ribosome is much more favorable than that of ester aminolysis in solution. Consequently, Sievers et al. proposed that the rate of peptide formation was enhanced mainly by the correct positioning of the substrates and/or water exclusion from the PTC. They considered this to be a non-conventional catalytic effect and called it an “entropic trap” [30].

**Figure 1:**
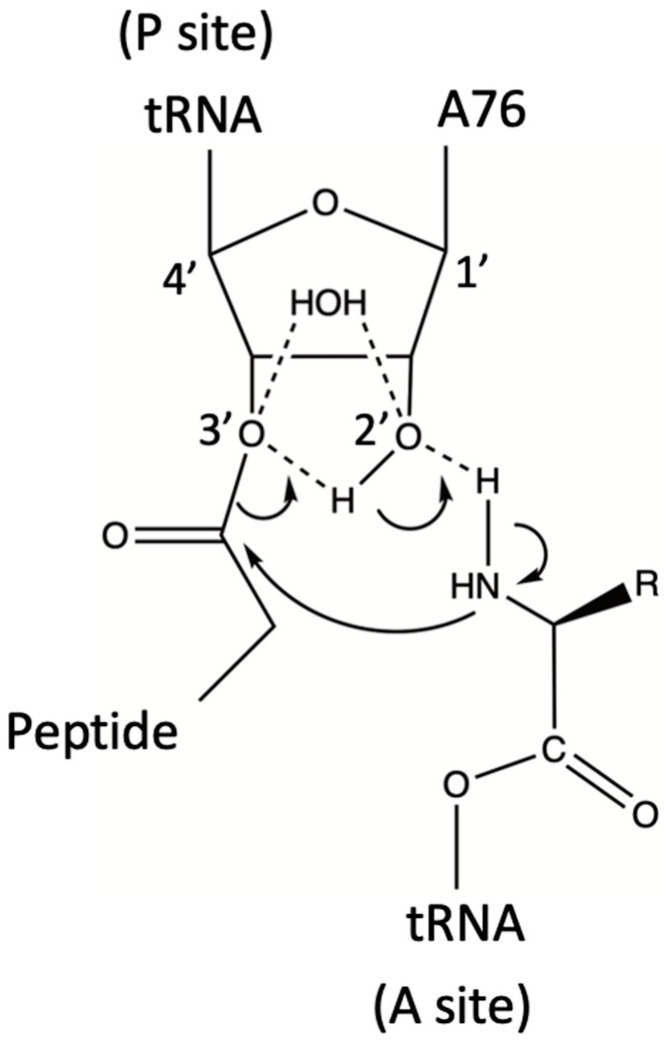
The ribosome promotes peptide synthesis through substrate-assisted catalysis and a low entropy of activation. The transition state involves a hexagonal cycle composed of –3′O–H–2′O–H–N–C(=O) (Scheme 2A in ref. [31]).

Also in 2004, and based on previous work by Hansen et al. [32], Weinger and coworkers performed the in silico combination of crystal structures of the ribosome with substrates bound to either the P- or the A-site of the PTC [33]. In these mixed models, the tRNA holding the nascent polypeptide chain was bound to the P-site, whereas the aminoacylated tRNA was bound to the A-site. This structural arrangement had the –NH_2_ group of the latter poised at the correct distance and angle to attack the C atom of the activated ester bond, which linked the A76 ribose ring of the P-bound tRNA to the nascent polypeptide chain (Figure 1). The in silico-combined structure also strongly suggested that only the ribosyl 2′ OH of the 3′ A76 from the peptidyl-tRNA could form a hydrogen bond with the attacking -NH_2_ group [33]. Such an H-bond is necessary to render the N atom nucleophilic enough to react with the ester carbon atom and form the required C-N peptide bond. This observation was very important because, although initial models had considered that the PTC could mediate peptide synthesis by acid-base catalysis [32], the replacement of several proposedly active ribosomal groups had only a minimal impact on that process [34,35,36].

Next, Weinger et al. performed a series of studies where the 2′ OH group of the P-site tRNA A76 ribose was replaced by either 2′ H or 2′ F [33]. Although the binding of the modified substrates was not affected, there was a 10^6^-fold reduction in the rate of peptide bond formation. This result confirmed the central role of the 2′ OH function in catalysis because it was the only functional group close to the ribosomal active site, the removal of which significantly affected the PTC rate. It also indicated that the “entropic trap” is not the only factor that determines the rate enhancement of peptide synthesis by the ribosome. Weinger and co-workers referred to the P-site A76 2′ OH-dependent mechanism as “substrate-assisted catalysis” (SAC) [33]. In a later paper, Changalov et al. proposed that the ribosyl 2′ OH group can also act as a general acid during a synthetic reaction by transferring one of the amino protons to the leaving tRNA A76 3′-oxyanion [31] (Figure 1).

Two theoretical papers published in 2005, respectively, discussed the results published by Sievers et al. [30] and Weinger et al. [33]. In one of these papers, Trobro & Aqvist reported a series of simulations concerning the reactant and tetrahedral intermediate states of peptide synthesis. Their calculations suggested the existence of a ribosomal preorganized H-bond network, already poised for catalysis [37]. The simulations also depicted an intra-reactant proton transfer pathway via the 2′ OH group of A76 after the attack of the A-site aminoacyl –NH_2_ group on the P-site ester (Figure 1). Thus, and as also proposed by Changalov et al. [31], during catalysis, the 2′ OH group functions as a proton shuttle that also involves the 3′ OH group [37]. The corresponding calculated rate enhancement was about 10^5^, and the catalytic effect was considered to be entirely entropic; it involved the reduction in solvent reorganization without resorting to alignment or “proximity/propinquity” effects. The authors concluded that the ribosome is an ancient catalyst that plays according to rules that are chemically different from those of enzymes [37]. Nevertheless, the structural preorganization of the active site is a common feature of the two classes of catalysts. This work was overall supportive of the thermodynamic conclusions reported by Sievers et al. [30].

The other theoretical paper to be discussed here was authored by Sharma et al., who analyzed both SAC and proximity/propinquity effects on ribosomal peptide synthesis [38]. These authors carried out a systematic evaluation of the energetics of the ribosyl 2′ OH-assisted catalytic mechanism in solution and compared it with the SAC proposed by Weinger et al. [33] (Figure 1). They concluded that the proximity/propinquity factor is small and that a large part of the catalytic effect is due to a drop in the solvation entropy. Furthermore, Sharma et al. proposed that the required reduction in the free energy of activation is due to electrostatic effects [38]. Although it was difficult to divide the activation free energy into its entropic and enthalpic components, these authors considered that the fundamental role assigned to SAC in ribosomal catalysis [33] may be an oversimplification: their calculations showed that the substrate-assisted reaction in water was not faster than the corresponding water-assisted reaction in water. Conversely, in the PTC, and thanks to the local electrostatic fields, the reaction should be assisted by the A76 ribosyl 2′ OH. Thus, like in many modern enzymes, electrostatic and preorganization effects are present in the ribosome, and they would have been incorporated early in its structure [38].

It is also interesting to note that, in these studies, the “substrate juxtaposition” factor proposed earlier [28], which intuitively could be considered a determinant for catalysis, appears to be thermodynamically less important than what might be expected.

Taken together, the above results portray a rather complex scenario for the origin of ribosomal peptide synthesis. On the one hand, both the “entropic trap” and “substrate-assisted catalysis” concepts are consistent with a “simpler-than-the-extant-ribosome” catalytic RNA molecule; this proto-ribosome would have already facilitated the attack of the N atom from an aminoacylated proto-tRNA to the activated peptidyl ester C atom of another proto-tRNA. On the other hand, if electrostatic and preorganization effects are also essential, then a more complex model might be required to explain primordial peptide synthesis. However, one important point to consider here is that, in their simulations, Sharma et al. compared the reaction at the PTC with the corresponding reactions in water [38]. There are now reasons to speculate that pre-Last Universal Common Ancestor (pre-LUCA) metabolic pathways were subjected to atmospheric/local variations that would have significantly modified the electrostatic environment of the proto-ribosome through dry/wet cycles. In fact, these cycles have been considered to be essential for the evolution of the metabolism and proto-cells [39,40]. The changes provoked by varying water activity levels would have facilitated, through natural selection, (i) primordial coded peptide synthesis and translocation and (ii) the emergence of electrostatics and preorganization in the proto-ribosome PTC. Since the target ester carbonyl carbon of the peptidyl-tRNA substrate is electrophilic, the only other requirement for the substitution reaction to occur is an unprotonated, nucleophilic α–NH_2_ group. Under the appropriate conditions, the amide C-N bond formation from the reaction of an amine with an activated carboxylate has already been observed on dehydrated TiO_2_ and silica surfaces [41,42].

Youngman et al. have described the ribosomal PTC site as being composed of an “inner” and an “outer” layer of conserved nucleotides relative to the catalytic site [34]. Interestingly, they found that inner nucleotides are not involved in catalysis; instead, they participate in peptide hydrolysis and release. Conversely, the outer-layer nucleotides would orient the tRNA substrates for peptide bond formation [34]. It seems unlikely that both activities could have appeared at the same time, and, certainly, polypeptide release should have evolved after peptide synthesis. Therefore, peptide translocation could have been preceded by a simpler mechanism, where the adaptor RNA carrying the nascent polypeptide chain alternated unbinding and rebinding to the catalytic RNA, as the chain grew, eventually detaching itself from the adaptor through hydrolysis.

There is some experimental basis to propose that the earliest coded peptide synthesis was carried out by RNAs that were rudimentally multifunctional. Indeed, tRNAs and rRNAs have significant nucleotide sequence homologies scattered throughout [43,44], and the high frequency of potential triplets found in 144 tRNAs suggests that they are related to early genes [45].

### 3.2. Aminoacyl-tRNA Synthetases

Aminoacyl-tRNA synthetases (aa-RSs) are considered to be very old enzymes [46]. There are two classes of aa-RSs called I and II [47]. Although they have similar catalytic mechanisms, there are also some significant differences [46]. Class I aa-RSs acylate tRNAs exclusively at the ribosyl 2′ OH, whereas Class II aa-RSs, with the exception of Phe-RS, acylate only at the 3′ OH group [48]. Because only 3′-aminoacylated tRNAs can react at the PTC (Figure 1), transacylation of the 2′-aminoacylated tRNAs to the 3′ position is required for peptide synthesis [48].

There are several observations that suggest that primordial Class I and II aa-RSs were coded by the complementary strands of an RNA duplex [21,49,50]. We have proposed a way in which this process could have originated [51], which is based on Yarus’ notion of stereochemical anticodon–amino acid binding [17] and on peptide amide bond formation by amines and activated carboxylates on a mineral surface [41,42]. As it has already been proposed (see Section 2), one of the RNA duplex strands could have generated, through direct binding and polymerization, a coded polypeptide with some functional capabilities. After melting, a process required for RNA replication, this strand could have remained bound to that polypeptide and, consequently, it would have been stabilized by it. However, if the opposite strand did not interact with a similarly stabilizing polypeptide, its subsequent replication would be compromised. Hence, the most effective way for a duplex RNA to evolve would be to have both strands coding for stabilizing peptides (which, eventually, would become full-fledged enzymes) [51].

Class I aa-RSs active sites adopt a Rossmann nucleotide-binding fold, whereas Class II aa-RSs have a corresponding antiparallel β-strand motif. The Rossmann fold is considered to be very ancient, having predated LUCA [52,53]. Consequently, it is tempting to speculate that it could have first appeared in primordial Class I aa-RSs. The reaction of both classes of aa-RSs is divided into two steps, (a) ATP-driven amino acid activation and (b) amino acid transfer:

(a) aa + ATP ↔ aa-AMP + PP_i_
(b) aa-AMP + tRNA^aa^ ↔ aa-tRNA^aa^ + AMP

**Figure 2:**
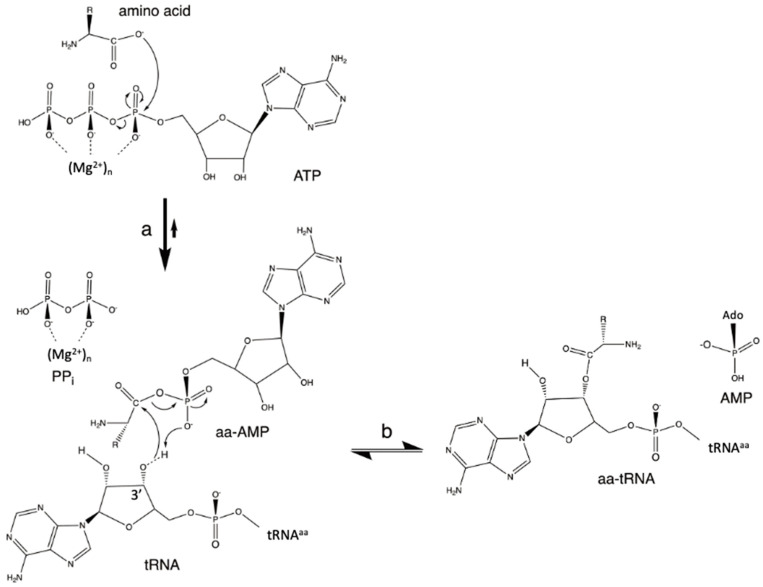
Synthesis of an aminoacylated-tRNA by a Class II aa-RS: (a) amino acid activation by phosphorylation; and (b) aminoacylation of the tRNA (figure adapted from ref. [46]).

Reaction (a) in Figure 2 shows that aa-AMP formation is made possible by Mg^2+^ coordination to the ATP triphosphate chain. This coordination is essential to render the P atom of the α-phosphate group electrophilic enough for its S_N_2-like reaction with the α-carboxylic O^−^ atom of the amino acid. Remarkably, the reactivity of ATP—and other nucleotides (NTPs)—towards a nucleophilic attack is very often directly modulated by positive counterions within proteins [54]. The interpretation of X-ray crystal structures of several aa-RSs and the corresponding mutagenesis studies have led to the conclusion that, in both aa-RS classes, the base required in reaction (b) is not supplied by the protein but by the substrate [55].

As shown in Figure 2b, in the Class II aminoacylation mechanism, one of the aa-AMP nonbridging phosphate oxygen deprotonates the ribosyl 3′ OH of the 3′ A76 from the tRNA^aa^.

The resulting nucleophilic O^−^ atom attacks the adenylate carbonyl carbon, and the C-O bond breaks, liberating AMP. Thus, as in polymerases (see below), there is an SAC process involving this 3′ O atom. Furthermore, both PPi and AMP are good leaving groups [46].

### 3.3. DNA-Dependent RNA Polymerases

**Figure 3:**
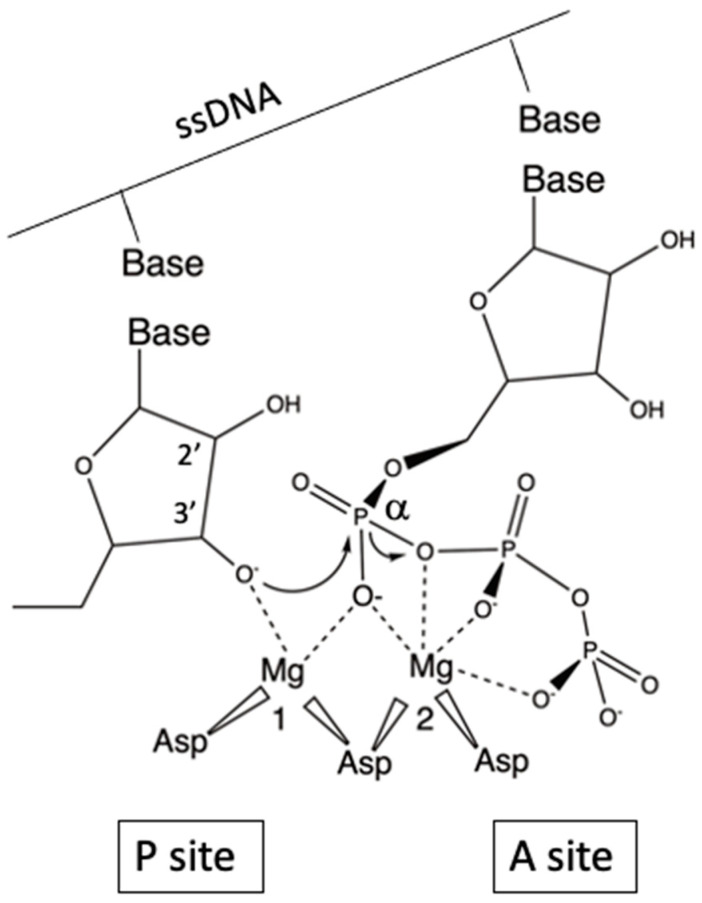
DNA-dependent RNA polymerase reaction. This figure was adapted from ref. [57].

A third early fundamental reaction relevant to the points being made in this review is the polymerization of NTPs to form RNAs. DNA- and RNA-dependent polymerases have similar folding and catalytic mechanisms [56]. Here, we will discuss the case of DNA-dependent RNA polymerases. Figure 3 depicts the general S_N_2 mechanism of nucleotide addition. The ssDNA strand serves as a template for the synthesis of a complementary ssRNA strand through the respective Watson–Crick T-A/A-U and C-G/G-C pairings. The reaction is made possible by (i) Mg^2+^ coordination to the α-phosphate of the incoming NTP (Mg1 and Mg2, A-site) and (ii) the deprotonation of the ribosyl 3′ OH group of the nascent RNA (Mg1, P-site). An electrophilic Pα atom and a nucleophilic O^−^ atom are, thus, well poised for the S_N_2 reaction to take place and form a bridging P–O bond.

It is also interesting to note that, in reactions catalyzed by these polymerases, the acid-base protein component does not seem to be determinant. Instead, the reactions would mostly depend on positional catalysis [56], which might also be defined as an “entropic trap”. In this respect, they resemble the reaction catalyzed by the ribosome (Figure 1). In addition, although this may not be considered to be a proper case of SAC (because the 3′ OH is not deprotonated by a substrate but by a Mg^2+^ ion), there are clear similarities between this reaction and aa-AMP synthesis. These similarities include the following: (i) the neutralization of the α-phosphate negative charge by Mg^2+^ ions; (ii) the lack of a direct involvement of protein residues in the catalysis; (iii) the stereochemistry of the nucleophilic substitution reaction; and (iv) the release of PPi (see Figure 2a and Figure 3).

## 4. Conclusions

In modern biology, the three reactions described above are connected in the following sequence (where “pol” is polymerase, “RS” RNA aminoacyl synthetase, and “aa” amino acid):


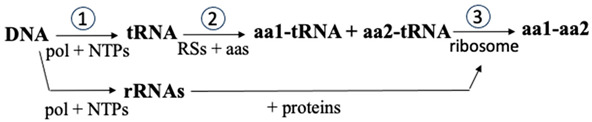


As highlighted above, a remarkable feature of these reactions is that they do not require the direct catalytic involvement of either RNA or protein residues, which, instead, play structural and/or electrostatic roles. In each case, a function of one of the substrates, either the P atom of a Mg^2+^-neutralized phosphate group or the carbonyl carbon of an ester or that of a phosphorylated species, is already electrophilic enough to be attacked by a suitable nucleophile. Consequently, in this series of reactions, substrate-assisted catalysis concerns either the stabilization or the generation of such a nucleophile. As previously shown, the latter can be the O^−^ atom of the aminoacyl carboxylate of a free amino acid, the neutral –NH_2_ group of an aa-tRNA H-bonded to a ribosyl 2′ OH group, or the deprotonated 3′ OH group of either a tRNA or a nascent RNA strand.

There are other examples of SAC involving a phosphate group neutralized by divalent cations [58]. For instance, Jeltsch et al. have reported that, in DNA endonucleases, the substrate phosphate group 3′ to the scissile bond serves to deprotonate an attacking water molecule [59]. This type of mechanism has also been predicted to function in GTPases [60,61] and in self-cleaving group-I intron ribozymes [62]. As in reaction (b) of aa-RSs (Figure 2), in all these other cases, a phosphate group acts as a general base, extracting a proton to generate an attacking –O^−^ nucleophile.

The combination of the entropic trap effect with SAC [33] would match the expected limited possibilities of a primordial catalyst. Indeed, being able to constructively position two activated substrates, which, furthermore, would help to catalyze their own reaction, should be evolutionary simpler than having an active site already equipped with specifically positioned functional groups.

Taken together, these observations allow for a plausible description of an initial period of life evolution when proto-metabolic reactions took place on, or close to, mineral surfaces that underwent wet/dry cycles. Under these conditions, nucleotides and amino acid would have polymerized when the water activity was very low (the “dry” period) and would have diffused to reach different mineral environments during the “wet” period. The crucial generation of electrophiles, such as the carboxyl C atom and the phosphate P atom, could have been carried out by mineral superficial Lewis acids, such as the Ti^4+^ ion of TiO_2_ [42] or the SiO_2_/SiO_3_-strained ring defects on a silica surface [41]. In modern aa-RSs and polymerases, the substrate phosphate groups interact with contiguous Mg^2+^ ions in an orientation that is reminiscent of the one they might have adopted when binding positively charged functions on a mineral surface. It is also interesting to note that, besides Mg^2+^, the Cys-RS from *Escherichia coli* uses an active site Zn^2+^ to recognize the –SH group of Cys while rejecting the similar Ser and Ala amino acids [46]. Conversely, in Thr-RS, an active site Zn^2+^ ion critically selects Thr against the similar Val and Ala (but less well against Ser). Val does not have a metal-ligand function, and the S atom from Cys may be too large for proper coordination [46]. Thus, different charges on, or close to, different mineral surfaces might have also helped in discriminating between amino acids during the early stages of protein synthesis evolution.

As the proto-cell evolved and acquired a semipermeable membrane, it would have detached itself from the mineral surface [26], and, consequently, it would have become permanently exposed to relatively high amounts of water activity. At this point, phosphate, thanks to its electron-withdrawing properties when neutralized, would have replaced the mineral Lewis acids. Furthermore, thanks to its dehydrating power, it would have made condensation reactions possible under relatively high water activity levels through ATP-mediated phosphorylation [63]. This is clearly illustrated here by the fact that, although a peptide bond formation is a condensation, i.e., it is formally a dehydrating reaction, no water is released in any of the above reactions. This observation underscores the very important dehydrating role of phosphate in biology [63,64].

The SAC reactivities of ribosyl 2′ OH as a base (ribosome) and deprotonated 3′ OH as a nucleophile (aminoacyl RS and RNA polymerase) are remarkable features of these putatively early reactions. Among the four sugar pentoses, namely, ribose, arabinose, xylose, and lyxose, only the β-d-ribose furanose ring has a 2′-3′ *cis*-diol *trans* to the groups bound to the C1′ and C4′ positions. Besides having this catalytic *cis*-diol well suited for the PTC reaction, β-d-ribose is also the only pentose that will allow the essential free rotation of the –OH, phosphate, and nucleobase groups in nucleotides [65]. Interestingly, Akouche et al. showed that β-d-ribose can be stabilized on a fumed silica surface [66]; and, as mentioned above, they were also able to sequentially synthetize PRPP from phosphate and ribose; furthermore, by adding adenine, they also obtained AMP on the same mineral support [12].

In the contemporary metabolism, d-ribose is exclusively synthesized in the pentose phosphate pathway as its 5-phosphate adduct (R5P), which is then phosphorylated to give PRPP. As discussed by Keller et al., R5P is five times more stable than other pentose phosphate intermediates, and its formation was found to be the fastest in an iron-rich simulated Archean ocean reaction medium [67]. Thus, the results reported by Akouche et al. [66] could be of significant relevance to understanding the initial association of ribose and phosphate and their subsequent role in genetics and peptide bond synthesis.

**Figure 4:**
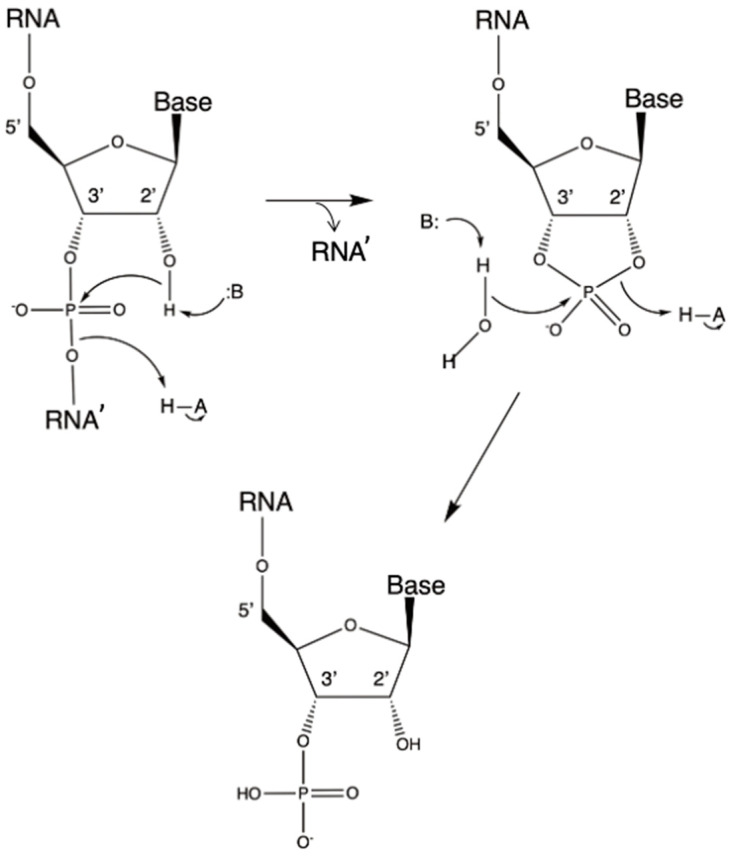
Common RNA hydrolysis requires both a base (B) and a proton donor (H–A).

It is, however, also important to consider that, under basic conditions, a deprotonated ribosyl 2′ OH is deleterious, as it leads to RNA hydrolysis (Figure 4), which must have posed a stability problem in early life evolution.

Given the central role of the ribosyl 2′ OH in peptide synthesis (Figure 1) and in spite of being a source of RNA instability, its elimination to yield deoxyribose and the more stable DNA polymer should have occurred when that synthetic path was already well established. Indeed, contemporary ribonucleotide reductases (RRs), responsible for this process, use a radical-based reaction [68], which, given its complexity, would have been hardly accessible to both catalytic RNAs and simple proto-enzymes. Of the three types of RRs known, the strictly anaerobic type III, which consists of two homodimeric proteins, NrdG and NrdD, is, most likely, the ancestral form [69]. NrdG uses the S-adenosylmethionine (SAM) cofactor and a [4Fe–4S] cluster to generate a glycyl radical in NrdD. dNTPs are generated by the latter protein using the formate → CO_2_ reaction as an electron donor and ATP as an allosteric effector [69]. The stereochemistry of the reaction is complex, as several radical species are involved, and the 2′ OH has to be selectively eliminated.

On page 3, we enumerated the major factors that contribute to catalysis in modern enzymes. Of these, only the entropy and propinquity effects are clearly also found in the three reactions described in this review. The rest of them represent evolutionary improvements in biocatalysis. It is worth mentioning in passing that the proposed early catalytic characteristics of the above reactions are also found in bacterial quinolinate synthase, the key enzyme of nicotinamide adenine dinucleotide (NAD) synthesis, which has three Rossmann fold-like domains which provide coordinating Cys residues to a [4Fe–4S] cluster, leaving a reactive unliganded Fe ion in its active site [70]. Interestingly, both substrates, iminoaspartate and dihydroxyacetone phosphate, are already activated for the first step in the nucleophilic substitution reaction, which does not involve catalytically active residues from the enzyme. In the second step, the intermediate species undergoes an intramolecular cyclization reaction that involves the nucleophilic attack of an -NH_2_ group to the carbonyl carbon of an aldehyde rendered electrophilic enough through its O-binding to the unique cluster Fe [70]. The similarities with the reactions analyzed in this review are evident. In addition, the activation of the carbonyl function by the unique Fe ion is reminiscent of similar reactions on mineral surfaces [41,42].

In conclusion, the analysis of the three reactions sheds significant light on the natural selection of both ribose and phosphate as essential primordial elements of the genetic machinery through their substrate-assisted catalysis. If life exists elsewhere in the universe, it will most likely have a genetic system, the components of which will share the properties of ribose and phosphate, both in their substrate-assisted reactions and as part of informational macromolecules.
